# Comprehensive analysis of diverse low-grade neuroepithelial tumors with *FGFR1* alterations reveals a distinct molecular signature of rosette-forming glioneuronal tumor

**DOI:** 10.1186/s40478-020-01027-z

**Published:** 2020-08-28

**Authors:** Calixto-Hope G. Lucas, Rohit Gupta, Pamela Doo, Julieann C. Lee, Cathryn R. Cadwell, Biswarathan Ramani, Jeffrey W. Hofmann, Emily A. Sloan, Bette K. Kleinschmidt-DeMasters, Han S. Lee, Matthew D. Wood, Marjorie Grafe, Donald Born, Hannes Vogel, Shahriar Salamat, Diane Puccetti, David Scharnhorst, David Samuel, Tabitha Cooney, Elaine Cham, Lee-way Jin, Ziad Khatib, Ossama Maher, Gabriel Chamyan, Carole Brathwaite, Serguei Bannykh, Sabine Mueller, Cassie N. Kline, Anu Banerjee, Alyssa Reddy, Jennie W. Taylor, Jennifer L. Clarke, Nancy Ann Oberheim Bush, Nicholas Butowski, Nalin Gupta, Kurtis I. Auguste, Peter P. Sun, Jarod L. Roland, Corey Raffel, Manish K. Aghi, Philip Theodosopoulos, Edward Chang, Shawn Hervey-Jumper, Joanna J. Phillips, Melike Pekmezci, Andrew W. Bollen, Tarik Tihan, Susan Chang, Mitchel S. Berger, Arie Perry, David A. Solomon

**Affiliations:** 1 Division of Neuropathology, Department of Pathology, University of California, San Francisco, 513 Parnassus Avenue, Health Sciences West 451, San Francisco, CA 94143 USA; 2 Institute for Human Genetics, University of California, San Francisco, San Francisco, CA USA; 3 Departments of Pathology, Neurology, and Neurosurgery, University of Colorado, Aurora, CO USA; 4 Department of Pathology, Sutter Medical Center, Sacramento, CA USA; 5 Department of Pathology, Oregon Health & Science University, Portland, OR USA; 6 Division of Neuropathology, Department of Pathology, Stanford University, Palo Alto, CA USA; 7 Department of Anatomic Pathology, University of Wisconsin-Madison, Madison, WI USA; 8 Division of Hematology, Oncology, and Bone Marrow Transplant, Department of Pediatrics, University of Wisconsin-Madison, Madison, WI USA; 9 Department of Pathology, Valley Children’s Hospital, Madera, CA USA; 10 Department of Hematology/Oncology, Valley Children’s Hospital, Madera, CA USA; 11 Department of Pediatric Oncology, Dana Farber Cancer Institute, Boston, MA USA; 12 Department of Pathology, UCSF Benioff Children’s Hospital Oakland, Oakland, CA USA; 13 Department of Pathology, University of California, Davis, Sacramento, CA USA; 14 Division of Pediatric Hematology/Oncology, Department of Pediatrics, Nicklaus Children’s Hospital, Miami, FL USA; 15 Department of Pathology, Nicklaus Children’s Hospital, Miami, FL USA; 16 Department of Pathology, Cedars-Sinai Medical Center, Los Angeles, CA USA; 17 Division of Pediatric Hematology/Oncology, Department of Pediatrics, University of California, San Francisco, San Francisco, CA USA; 18 Department of Neurology, University of California, San Francisco, San Francisco, CA USA; 19 Division of Neuro-Oncology, Department of Neurological Surgery, University of California, San Francisco, San Francisco, CA USA; 20 Department of Neurological Surgery, University of California, San Francisco, San Francisco, CA USA

**Keywords:** Rosette-forming glioneuronal tumor (RGNT), Extraventricular neurocytoma (EVN), Dysembryoplastic neuroepithelial tumor (DNT), Pilocytic astrocytoma, *FGFR1*, *PIK3CA*, *PIK3R1*, Molecular neuropathology, DNA methylation profiling

## Abstract

The *FGFR1* gene encoding fibroblast growth factor receptor 1 has emerged as a frequently altered oncogene in the pathogenesis of multiple low-grade neuroepithelial tumor (LGNET) subtypes including pilocytic astrocytoma, dysembryoplastic neuroepithelial tumor (DNT), rosette-forming glioneuronal tumor (RGNT), and extraventricular neurocytoma (EVN). These activating *FGFR1* alterations in LGNET can include tandem duplication of the exons encoding the intracellular tyrosine kinase domain, in-frame gene fusions most often with *TACC1* as the partner, or hotspot missense mutations within the tyrosine kinase domain (either at p.N546 or p.K656). However, the specificity of these different *FGFR1* events for the various LGNET subtypes and accompanying genetic alterations are not well defined. Here we performed comprehensive genomic and epigenomic characterization on a diverse cohort of 30 LGNET with *FGFR1* alterations. We identified that RGNT harbors a distinct epigenetic signature compared to other LGNET with *FGFR1* alterations, and is uniquely characterized by *FGFR1* kinase domain hotspot missense mutations in combination with either *PIK3CA* or *PIK3R1* mutation, often with accompanying *NF1* or *PTPN11* mutation. In contrast, EVN harbors its own distinct epigenetic signature and is characterized by *FGFR1*-*TACC1* fusion as the solitary pathogenic alteration. Additionally, DNT and pilocytic astrocytoma are characterized by either kinase domain tandem duplication or hotspot missense mutations, occasionally with accompanying *NF1* or *PTPN11* mutation, but lacking the accompanying *PIK3CA* or *PIK3R1* mutation that characterizes RGNT. The glial component of LGNET with *FGFR1* alterations typically has a predominantly oligodendroglial morphology, and many of the pilocytic astrocytomas with *FGFR1* alterations lack the biphasic pattern, piloid processes, and Rosenthal fibers that characterize pilocytic astrocytomas with *BRAF* mutation or fusion. Together, this analysis improves the classification and histopathologic stratification of LGNET with *FGFR1* alterations.

**Electronic supplementary material:**

The online version of this article (10.1186/s40478-020-01027-z) contains supplementary material, which is available to authorized users.

## Introduction

Classification of glial and glioneuronal neoplasms is an evolving process that is no longer exclusively based on the combination of radiographic and histologic features, but also now includes both genetic and epigenetic signatures to facilitate the most accurate subtyping and prognostication [7, 13, 20, 29, 35]. One challenge that has arisen in terms of CNS tumor classification is that many genetic alterations are not specific to individual tumor entities, but can be seen across a multitude of different tumor types. For example, *IDH1/2* mutation is present in both diffuse astrocytic neoplasms and oligodendroglial neoplasms in the cerebral hemispheres of young adults. As a second example, *BRAF* mutations or fusions are present in a diverse spectrum of neuroepithelial tumors, including ganglioglioma, pilocytic astrocytoma, and pleomorphic xanthoastrocytoma (PXA). As such, it is not possible to classify CNS tumors based solely on the presence of a single genetic aberration in most cases. The most accurate diagnostic classification incorporates assessment of any accompanying alterations, such as those commonly co-occurring with *IDH1/2* mutation (*e.g. TP53* and *ATRX* mutation in astrocytomas vs. *CIC*, *FUBP1*, and *TERT* promoter mutations in oligodendrogliomas) or with *BRAF* mutation/fusion (*e.g. CDKN2A* homozygous deletion in PXA versus intact *CDKN2A* alleles in ganglioglioma and pilocytic astrocytoma) [6, 29, 30, 34, 44].

The *FGFR1* gene on chromosome 8p11.23 has emerged as a recurrently altered oncogene in a diverse spectrum of primary glial and glioneuronal tumor entities including DNT [31, 33, 34, 40, 41], RGNT [14, 19, 22, 36], EVN [38], pilocytic astrocytoma [3, 18, 27, 34, 44], high-grade astrocytoma with piloid features [2, 32], and H3 K27M-mutant diffuse midline glioma [24]. Notably, *FGFR1* alterations are not commonly found in IDH-wildtype glioblastoma in adults, IDH-mutant astrocytoma, IDH-mutant and 1p/19q-codeleted oligodendroglioma, H3.3 G34-mutant diffuse hemispheric glioma, ganglioglioma, polymorphous low-grade neuroepithelial tumor of the young (PLNTY), papillary glioneuronal tumor, myxoid glioneuronal tumor, multinodular and vacuolating neuronal tumor, diffuse leptomeningeal glioneuronal tumor (DLGNT), central neurocytoma, and ependymomas of any location or subtype [5, 6, 8, 9, 16, 17, 23, 24, 26, 28, 29]. However, fusions involving other *FGFR* genes are recurrently found in PLNTY (mostly involving *FGFR2*) and IDH-wildtype glioblastoma in adults (mostly involving *FGFR3*, typically with *TACC3* as the fusion partner) [4, 10, 17, 39]. Therefore, while the identification of an *FGFR1* alteration in a CNS tumor of uncertain subtype may help to narrow the differential diagnosis and exclude certain tumor entities, this single genetic finding in and of itself does not enable precise classification. Our study sought to refine classification of low-grade neuroepithelial tumors (LGNET) harboring *FGFR1* alterations by investigating if the specific type of *FGFR1* alteration, accompanying genetic alterations, tumor location, and epigenetic signature can help to more accurately stratify these tumors.

## Methods

### Patient cohort and tumor samples

Thirty patients with LGNET harboring pathogenic *FGFR1* alterations were included in this study. All tumor specimens were fixed in 10% neutral-buffered formalin and embedded in paraffin. Pathologic review of all tumors was conducted by a group of expert neuropathologists (MP, AWB, TT, AP, and DAS). Tumor tissue was selectively scraped from unstained slides or punched from formalin-fixed, paraffin-embedded blocks using 2.0 mm disposable biopsy punches (Integra Miltex Instruments, cat# 33-31-P/25) to enrich for as high of tumor content as possible. Genomic DNA was extracted from this macrodissected formalin-fixed, paraffin-embedded tumor tissue using the QIAamp DNA FFPE Tissue Kit (Qiagen).

### Targeted next-generation sequencing

Targeted next-generation sequencing was performed using the UCSF500 Cancer Panel as previously described [15, 20, 29]. Genomic DNA was also extracted from a peripheral blood or buccal swab sample as a source of constitutional DNA for discrimination of somatic versus germline status of identified variants for seven of the patients (PA #1, PA #3, PA #4, PA #6, DNT #1, EVN #2, and uLGNET #5) using the QIAamp DNA Blood Midi Kit (Qiagen). Capture-based next-generation DNA sequencing was performed using an assay that targets all coding exons of 479 cancer-related genes, select introns and upstream regulatory regions of 47 genes to enable detection of structural variants including gene fusions, and DNA segments at regular intervals along each chromosome to enable genome-wide copy number and zygosity analysis, with a total sequencing footprint of 2.8 Mb (Additional file 1: Table S1). Multiplex library preparation was performed using the KAPA Hyper Prep Kit (Roche) according to the manufacturer’s specifications using 250 ng of sample DNA. Hybrid capture of pooled libraries was performed using a custom oligonucleotide library (Nimblegen SeqCap EZ Choice). Captured libraries were sequenced as paired-end 100 bp reads on an Illumina HiSeq 2500 instrument. Sequence reads were mapped to the reference human genome build GRCh37 (hg19) using the Burrows–Wheeler aligner (BWA). Recalibration and deduplication of reads was performed using the Genome Analysis Toolkit (GATK). Coverage and sequencing statistics were determined using Picard CalculateHsMetrics and Picard CollectInsertSizeMetrics. Single nucleotide variant and small insertion/deletion mutation calling was performed with FreeBayes, Unified Genotyper, and Pindel (Additional file 1: Table S2). Large insertion/deletion and structural alteration calling was performed with Delly (Additional file 1: Table S3). Variant annotation was performed with Annovar. Single nucleotide variants, insertions/deletions, and structural variants were visualized and verified using Integrative Genome Viewer. Genome-wide copy number and zygosity analysis was performed by CNVkit and visualized using Nexus Copy Number (Biodiscovery) (Additional file 1: Table S4).

### DNA methylation profiling

250 ng of genomic DNA from the 30 tumors was bisulfite converted using the EZ DNA Methylation kit following the manufacturer’s recommended protocol (Zymo Research). Bisulfite converted DNA was then amplified, fragmented, and hybridized to Infinium EPIC 850k Human DNA Methylation BeadChips following the manufacturer’s recommended protocol (Illumina). Raw data files (.idat) generated by the iScan instrument were processed in the R statistical environment (v3.6.0) using the minfi package (v1.30.0) [1]. The detection *p* value for each sample was computed. All samples had detection p values universally less than 0.05. Additional quality control was performed by calculating the median log (base2) intensities for methylated and unmethylated signals for each array. All samples had unmethylated and methylated median intensity values above 10 that were used for analysis. Beta density plots for all samples before and after normalization were also examined for quality control. Functional normalization with NOOB background correction and dye-bias normalization was performed [12, 42]. Probe filtering was performed after normalization. Specifically, probes located on sex chromosomes, containing nucleotide polymorphisms (dbSNP132 Common) within five base pairs of and including the targeted CpG site, or mapping to multiple sites on hg19 (allowing for one mismatch), as well as cross reactive probes were removed from analysis.

The DNA methylation profiles of the 30 tumors were assessed together with 907 reference tumors spanning 20 CNS tumor entities previously generated at DKFZ (listed in Additional file 1: Table S5). These included 78 A IDH (astrocytoma, IDH-mutant), 46 A IDH-HG (astrocytoma, IDH-mutant, high-grade), 21 ANA (high-grade astrocytoma with piloid features), 21 CN (central neurocytoma), 8 DLGNT (diffuse leptomeningeal glioneuronal tumor), 78 DMG-K27 (diffuse midline glioma, H3 K27M-mutant), 22 EVN (extraventricular neurocytoma), 41 GBM-G34 (diffuse hemispheric glioma, H3 G34-mutant), 56 GBM MES (glioblastoma, IDH-wildtype, mesenchymal subclass), 64 GBM RTK1 (glioblastoma, IDH-wildtype, RTK1 subclass), 143 GBM RTK2 (glioblastoma, IDH-wildtype, RTK2 subclass), 13 GBM RTK3 (glioblastoma, IDH-wildtype, RTK3 subclass), 44 DNT (dysembryoplastic neuroepithelial tumor), 21 GG (ganglioglioma), 22 LGG MYB (low-grade glioma, MYB/MYBL1 fusion positive), 38 PA MID (pilocytic astrocytoma, midline subclass), 114 PA PF (pilocytic astrocytoma, posterior fossa subclass), 24 PA ST (pilocytic astrocytoma, supratentorial subclass), 9 RGNT (rosette-forming glioneuronal tumor), 44 PXA (pleomorphic xanthoastrocytoma). Since the reference cohort contained methylation data generated using the Infinium Human Methylation 450k BeadChips, the approximately 450,000 overlapping CpG sites between the EPIC 850k and 450k BeadChips were used in the analysis. A beta value matrix with 389,282 CpG probes was used for all downstream analysis. Row-wise standard deviation was calculated for each probe across all samples, and the 32,502 most differentially methylated probes with standard deviation > 0.178 were selected. Uniform manifold approximation and projection (UMAP) was used for non-linear dimensionality reduction to cluster samples with similar CpG methylation patterns [25]. UMAP was performed using uwot package (v 0.1.7) with the following analysis parameters: min_dist = 0.01, spread = 4, n_neighbors = 100, metric = cosine. The UMAP plot was visualized with ggplot2 (v 3.2.0) [https://ggplot2.tidyverse.org/]. Clustering assignment derived from the UMAP analysis was based on spatial proximity to the DKFZ reference tumor cohorts, with those tumors remote from the reference clusters categorized as unclassifiable via this methodology.

## Results

### Clinical features

**Fig. 1:**
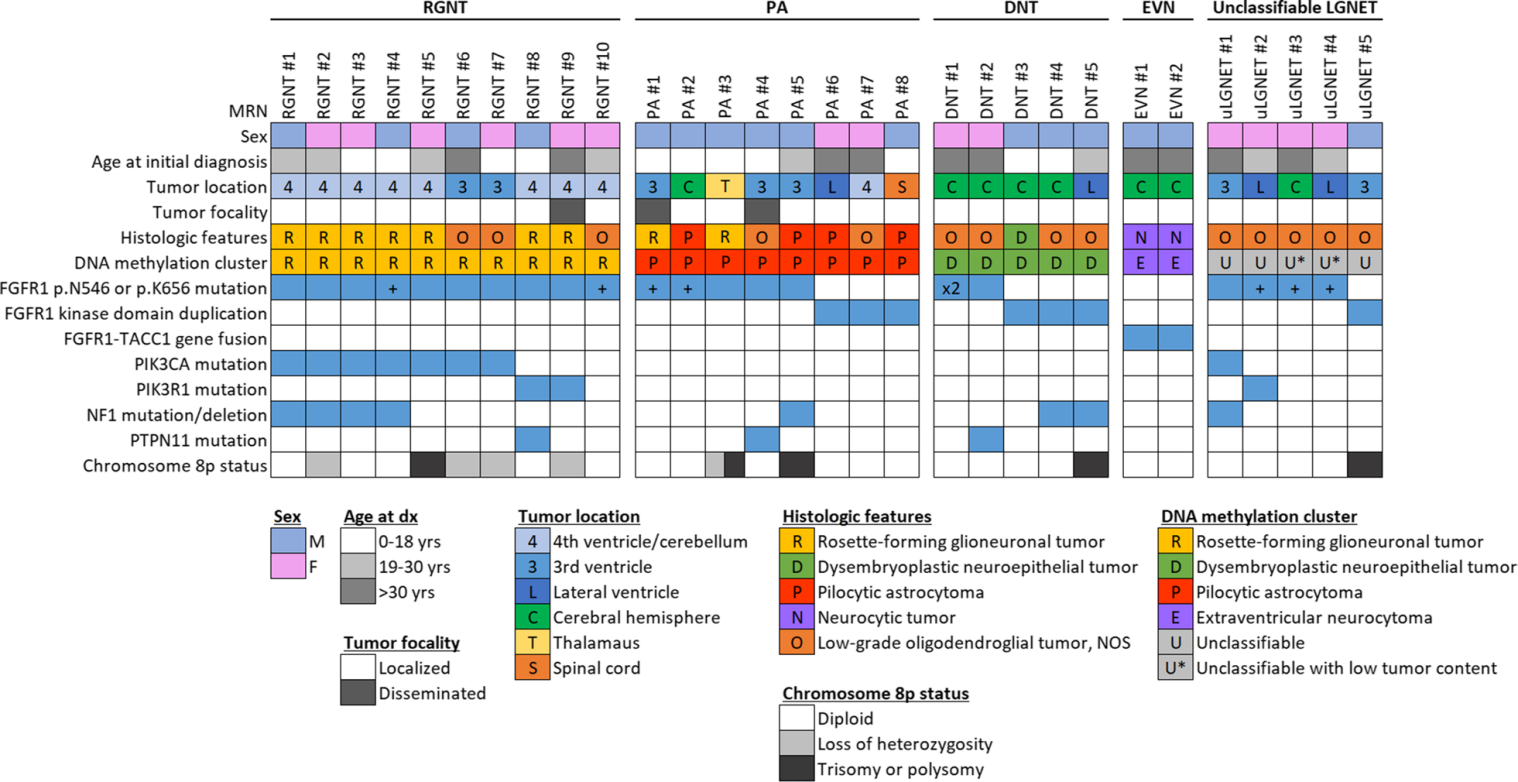
Oncoprint summary table of the clinical, histologic, genetic, and epigenetic features of the 30 low-grade neuroepithelial tumors (LGNET) with *FGFR1* alterations, grouped by methylation class derived from the UMAP clustering analysis. +, *FGFR1* secondary non-hotspot missense mutation. x2, *FGFR1* p.N546 and p.K656 hotspot mutations in combination

The study cohort included 30 patients with LGNET that had next-generation sequencing performed demonstrating an activating *FGFR1* alteration (Table 1 and Fig. 1). The cohort included 16 male and 14 female patients with a median age of 22 years at time of initial diagnosis (range 6–72 years). Tumors were centered in the cerebral hemispheres in 8 patients, the lateral ventricles in 4 patients, the third ventricle in 7 patients, the thalamus in 1 patient, the fourth ventricle or cerebellum in 9 patients, and the spinal cord in 1 patient.

**Table 1:** Summary of the cohort of 30 patients with low-grade neuroepithelial tumors harboring *FGFR1* alterations that were studied

Patient	Age at dx (yrs)	Sex	Tumor location	Histologic features	Methylation cluster	Genetic alterations	Chromosome 8p status
RGNT #1	26	M	4th ventricle/cerebellum	Rosette-forming glioneuronal tumor	RGNT	FGFR1 p.N546K (28%), PIK3CA p.H1047R (28%), NF1 p.K1444E (28%)	Diploid
RGNT #2	23	F	4th ventricle/cerebellum	Rosette-forming glioneuronal tumor	RGNT	FGFR1 p.N546K (42%), PIK3CA p.H1047R (29%), NF1 p.I2058V (44%)	LOH
RGNT #3	16	F	4th ventricle/cerebellum	Rosette-forming glioneuronal tumor	RGNT	FGFR1 p.N546K (43%), PIK3CA p.K111del (42%), NF1 p.E1206fs (40%)	Diploid
RGNT #4	14	M	4th ventricle/cerebellum	Rosette-forming glioneuronal tumor	RGNT	FGFR1 p.N546K (37%) + p.K523T (8%), PIK3CA p.E542K (22%), NF1 p.N2387_F2388del (30%)	Diploid
RGNT #5	30	F	4th ventricle/cerebellum	Rosette-forming glioneuronal tumor	RGNT	FGFR1 p.N546K (56%), PIK3CA p.H1047L (43%)	Trisomy
RGNT #6	46	M	3rd ventricle	Low-grade oligodendroglial tumor NOS	RGNT	FGFR1 p.N546K (55%), PIK3CA p.E542K (28%)	LOH
RGNT #7	11	F	3rd ventricle	Low-grade oligodendroglial tumor NOS	RGNT	FGFR1 p.K656E (86%), PIK3CA p.G106_E109del (32%)	LOH
RGNT #8	11	M	4th ventricle/cerebellum	Rosette-forming glioneuronal tumor	RGNT	FGFR1 p.K656E (29%), PIK3R1 p.T454_F456delinsT (26%), PTPN11 p.A72T (32%)	Diploid
RGNT #9	38	F	4th ventricle/cerebellum	Rosette-forming glioneuronal tumor	RGNT	FGFR1 p.N546K (77%), PIK3R1 p.L449_H450delinsF (39%)	LOH
RGNT #10	20	F	4th ventricle/cerebellum	Low-grade oligodendroglial tumor NOS	RGNT	FGFR1 p.K656E (37%) + p.D652G (37%)	Diploid
PA #1	13	M	3rd ventricle	Rosette-forming glioneuronal tumor	PA, PF	FGFR1 p.N546K (35%) + p.R675G (34%)	Diploid
PA #2	17	M	Cerebral hemisphere	Pilocytic astrocytoma	PA, MID	FGFR1 p.K656E (32%) + p.V561M (47%)	Diploid
PA #3	14	M	Thalamus	Rosette-forming glioneuronal tumor	PA, PF	FGFR1 p.N546K (69%)	Trisomy + LOH
PA #4	17	M	3rd ventricle	Low-grade oligodendroglial tumor NOS	PA, MID	FGFR1 p.N546K (40%), PTPN11 p.G60V (51%)	Diploid
PA #5	29	M	3rd ventricle	Pilocytic astrocytoma	PA, ST	FGFR1 p.K656E (32%), NF1 p.P866fs (24%)	Trisomy
PA #6	72	F	Lateral ventricle	Pilocytic astrocytoma	PA, ST	FGFR1 tandem duplication (p.V429_A815dup, exons 10-18)	Diploid
PA #7	46	F	4th ventricle/cerebellum	Low-grade oligodendroglial tumor NOS	PA, PF	FGFR1 tandem duplication (p.M390_D768dup, exons 9-18)	Diploid
PA #8	10	M	Spinal cord	Pilocytic astrocytoma	PA, PF	FGFR1 tandem duplication (p.V429_G791dup, exons 10-18)	Diploid
DNT #1	46	F	Cerebral hemisphere	Low-grade oligodendroglial tumor NOS	DNT	FGFR1 p.K656E (38%) + p.N546S (33%)	Diploid
DNT #2	42	F	Cerebral hemisphere	Low-grade oligodendroglial tumor NOS	DNT	FGFR1 p.N546K (30%), PTPN11 p.G503V (29%)	Diploid
DNT #3	6	M	Cerebral hemisphere	Dysembryoplastic neuroepithelial tumor	DNT	FGFR1 tandem duplication (p.V429_S785dup, exons 10-18)	Diploid
DNT #4	11	M	Cerebral hemisphere	Low-grade oligodendroglial tumor NOS	DNT	FGFR1 tandem duplication (p.V429_A815dup, exons 10-18), NF1 p.D1248fs (6%)	Diploid
DNT #5	22	M	Lateral ventricle	Low-grade oligodendroglial tumor NOS	DNT	FGFR1 tandem duplication (p.S420_K820dup, exons 9-18), NF1 p.P1421R (16%)	Trisomy
EVN #1	31	M	Cerebral hemisphere	Neurocytic tumor	EVN	FGFR1-TACC1 fusion (exons 1-18, exons 7-13)	Diploid
EVN #2	35	M	Cerebral hemisphere	Neurocytic tumor	EVN	FGFR1-TACC1 fusion (exons 1-17, exons 7-13)	Diploid
uLGNET #1	47	F	3rd ventricle	Low-grade oligodendroglial tumor NOS	Unclassified	FGFR1 p.N546K (49%), PIK3CA p.G118D (78%), NF1 exon 35 del	Diploid
uLGNET #2	26	F	Lateral ventricle	Low-grade oligodendroglial tumor NOS	Unclassified	FGFR1 p.K656E (29%) + p.I544V (31%), PIK3R1 p.K567E (28%)	Diploid
uLGNET #3	35	F	Cerebral hemisphere	Low-grade oligodendroglial tumor NOS	Unclassified	FGFR1 p.K656E (10%) + p.D652G (10%)	Diploid
uLGNET #4	21	F	Lateral ventricle	Low-grade oligodendroglial tumor NOS	Unclassified	FGFR1 p.K656E (6%) + p.D652G (6%)	Diploid
uLGNET #5	8	M	3rd ventricle	Low-grade oligodendroglial tumor NOS	Unclassified	FGFR1 tandem duplication (p.C389_G818dup, exons 9-18)	Polysomy (4N)

RefSeq transcript ID’s: FGFR1, NM_023110; PIK3CA, NM_006218; PIK3R1, NM_181523; NF1, NM_001042492; PTPN11, NM_002834

### Comprehensive genomic profiling results

Among the 30 LGNET from this cohort, 21 tumors were identified with kinase domain hotspot missense mutations (12 with p.N546K, 8 with p.K656E, and 1 with dual p.N546S and p.K656E), 7 tumors with kinase domain tandem duplication, and 2 tumors with *FGFR1*-*TACC1* in-frame gene fusion (Table 1, Fig. 1, Additional file 1: Tables S2 and S3). Among the 21 LGNET with either *FGFR1* p.N546 or p.K656 hotspot mutations, a second non-hotspot missense mutation in *FGFR1* was also identified in 8 tumors, which were uniformly present in cis (on the same allele) as the hotspot mutation when phasing was possible (n = 4). In six tumors, the *FGFR1* p.N546 or p.K656 mutation was present at an equal variant allele frequency as the second non-hotspot missense mutation, indicating that they were both acquired at a similar timepoint during tumorigenesis. In one tumor (RGNT #4), the *FGFR1* p.N546K mutation was present at 37% allele frequency whereas the second mutation (p.K523T) was subclonal and present at 8% allele frequency. In another tumor (PA #2), the *FGFR1* p.K656E hotspot mutation was present at 32% allele frequency whereas the second mutation (p.V561M) was present at an allele frequency of approximately 50%, suggestive of the latter non-hotspot mutation potentially being present in the germline and the former hotspot mutation likely being present as a somatic mutation acquired during tumor development. Notably, germline mutations in the *FGFR1* gene have been found in kindreds with familial occurrence of DNT [33].

Additional accompanying mutations were identified in a subset of the LGNET involving *PIK3CA* (n = 8), *PIK3R1* (n = 3), *NF1* (n = 8), and *PTPN11* (n = 3). No mutations, amplifications, deletions, or rearrangements were identified in this cohort of 30 LGNET in the following genes known to be important in the pathogenesis of glial and glioneuronal tumors: *ATRX*, *TERT* (including promoter region), *TP53*, *PPM1D*, *CDKN2A*, *CDK4*, *CDK6*, *RB1*, *BRAF*, *KRAS*, *MAP2K1*, *PRKCA*, *PDGFRA*, *EGFR*, *MET*, *NTRK1*-3, *FGFR2*, *FGFR3*, *ALK*, *ROS1*, *IDH1*, *IDH2*, *H3F3A*, *H3F3B*, *HIST1H3B*, *HIST1H3C*, *CIC*, *FUBP1*, *BCOR*, *BCORL1*, *MYB*, and *MYBL1*.

Chromosomal copy number analysis revealed that 13 of the LGNET had a balanced diploid genome without chromosomal gains, losses, or loss of heterozygosity (Additional file 1: Table S4). Among the remaining 17 LGNET, recurrent cytogenetic changes included trisomy 5 (n = 3), trisomy 6 (n = 7), trisomy 7 (n = 5), trisomy 11 (n = 5), and trisomy 12 (n = 8). Additionally, five tumors had trisomy (n = 4) or tetrasomy (n = 1) of chromosome 8, which includes the *FGFR1* locus and resulted in extra copies of the mutant or rearranged allele in tumor cells. Another four tumors had copy-neutral loss of heterozygosity of chromosome 8p, which includes the *FGFR1* locus and resulted in tumors with two copies of the mutant allele and zero copies of the wildtype allele. Two tumors had a focal amplification on chromosome 8p11.23 which included the *FGFR1* and *TACC1* loci and were the two tumors identified to harbor *FGFR1*-*TACC1* in-frame gene fusion. Associations between specific *FGFR1* alteration, accompanying genetic alteration(s), tumor histology, and epigenetic signature are described in detail below.

### Epigenomic clustering analysis

**Fig. 2:**
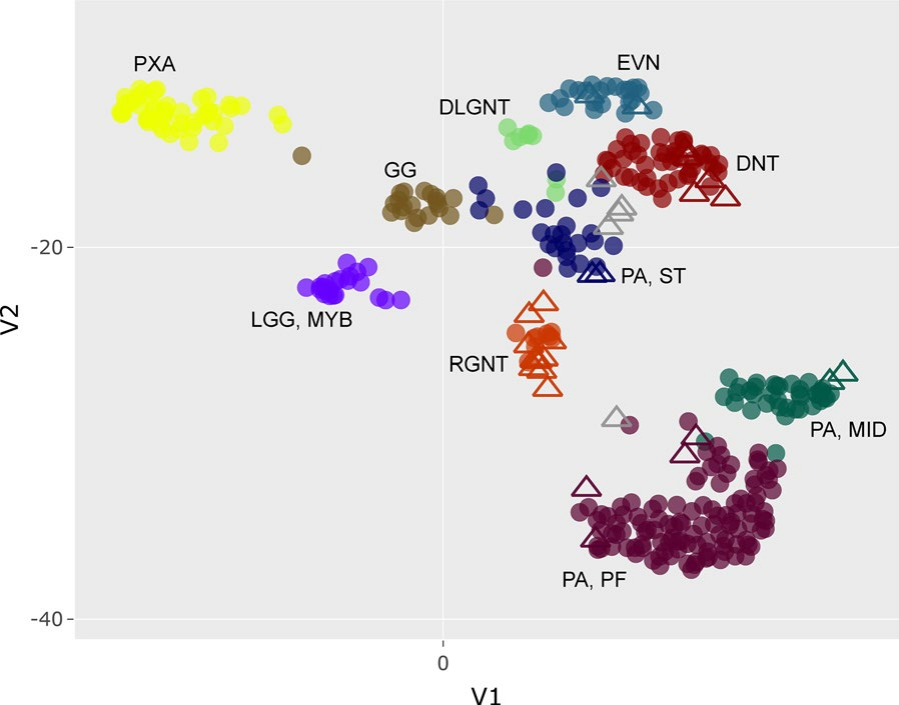
DNA methylation clustering analysis of the 30 LGNET with *FGFR1* alterations (triangles), alongside a reference set of CNS tumor samples generated at DKFZ (circles). Shown is a two-dimensional representation of pairwise sample correlations using the 32,000 most variably methylated probes by uniform manifold approximation and projection (UMAP). Five LGNET from this cohort did not definitively cluster with known reference classes and are colored gray (uLGNET #1–5). Reference methylation classes are: DLGNT, diffuse leptomeningeal glioneuronal tumor; DNT, dysembryoplastic neuroepithelial tumor; EVN, extraventricular neurocytoma; GG, ganglioglioma; LGG MYB, low-grade glioma with *MYB* or *MYBL1* rearrangement; PA MID, midline pilocytic astrocytoma; PA PF, posterior fossa pilocytic astrocytoma; PA ST, supratentorial/hemispheric pilocytic astrocytoma; PXA, pleomorphic xanthoastrocytoma; RGNT, rosette-forming glioneuronal tumor. See Additional file 1: Table S5 for the list of reference samples from DKFZ used in the clustering analysis

DNA methylation profiling was performed on the 30 LGNET with *FGFR1* alterations using the Infinium Human Methylation EPIC 850k BeadChip Arrays. The DNA methylation profiles of the 30 tumors were clustered together with 907 reference CNS tumors generated at DKFZ (Fig. 2, reference tumors listed in Additional file 1: Table S5) [7, 38]. Additionally, random forest classification using the DKFZ MolecularNeuropathology.org online classifier tool (version 11b4) was performed (Additional file 1: Table S6). The UMAP clustering analysis revealed that 25 of the 30 tumors closely clustered with known reference methylation classes: 10 with RGNT, 8 with pilocytic astrocytoma (2 with midline, 2 with supratentorial, and 4 with posterior fossa subclasses), 5 with DNT, and 2 with EVN. The remaining 5 tumors did not closely cluster with any of the known reference methylation classes. Notably, the predicted tumor content for two of these unclassifiable tumors was low, based on both microscopic assessment and also the *FGFR1* p.K656E mutant allele frequencies being 10% for uLGNET #3 and 6% for uLGNET #4. However, the predicted tumor content for the other three unclassifiable tumors was high, based on both microscopic assessment and *FGFR1* mutant allele frequencies greater than 25%.

By random forest classification using the DKFZ online classifier tool, only 12 of the 30 tumors were classifiable using a threshold calibrated score of greater than or equal to 0.9. There was agreement between the UMAP clustering analysis and the random forest prediction in each of these 12 cases. For the remaining 18 cases, the random forest classifier predicted a methylation class with a calibrated score between 0.3 and 0.9 in eight tumors, of which there was agreement with the UMAP clustering in five of these tumors. Notably, the current version 11b4 of the DKFZ online classifier tool does not include extraventricular neurocytoma (EVN) as a reference methylation class, accounting for two of the three cases in which there was discordance between the UMAP clustering analysis and random forest prediction. The remaining 10 cases had no methylation classes with calibrated score > 0.3 by random forest classification, which includes four of the five tumors which did not closely cluster with any reference methylation groups by UMAP analysis. Below we describe the clinical, histologic, and genetic features of the LGNET with *FGFR1* alterations aligning with each of the different methylation classes based on the UMAP clustering analysis.

### Rosette-forming glioneuronal tumor

**Fig. 3:**
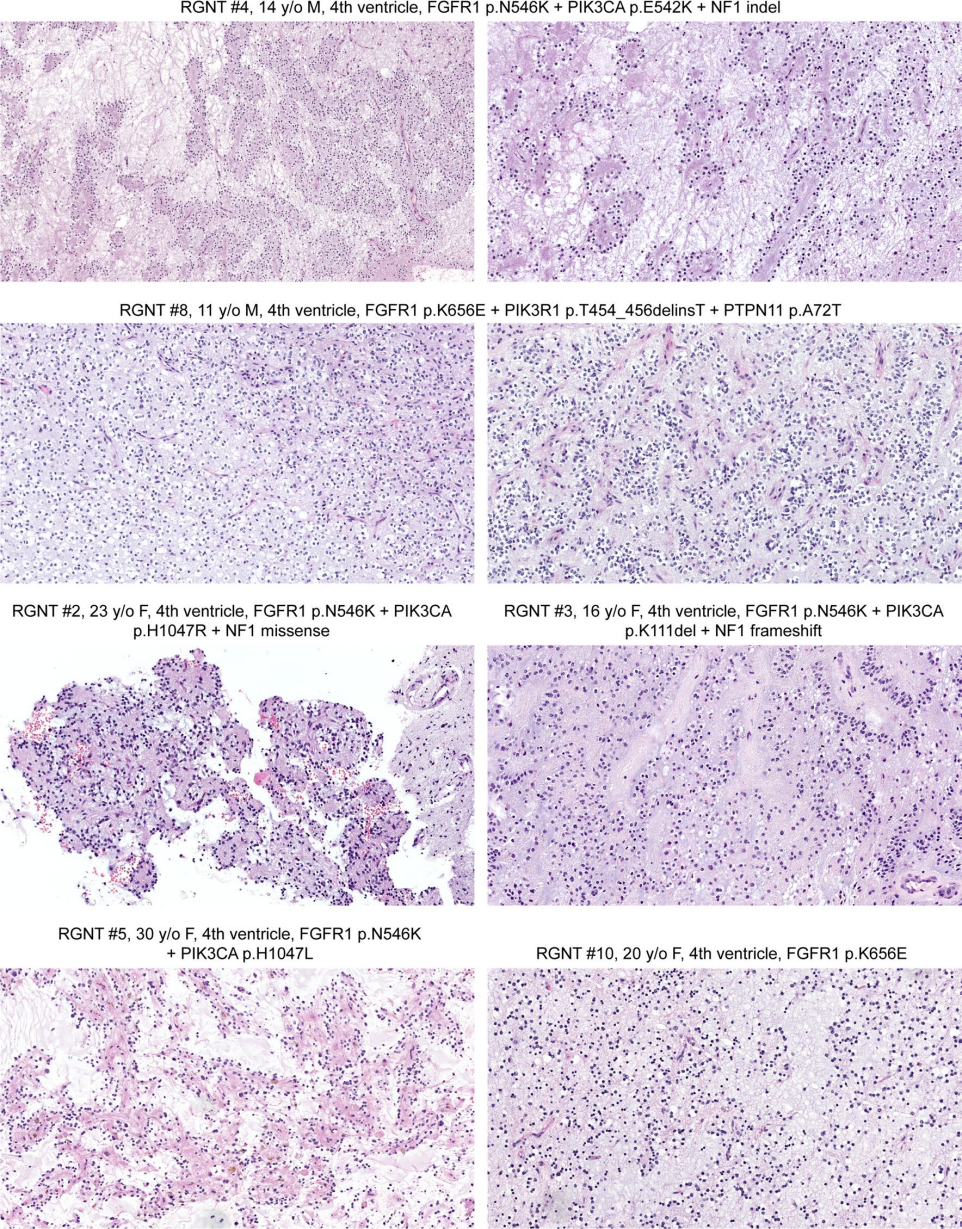
Histologic spectrum of epigenetically defined rosette-forming glioneuronal tumors harboring *FGFR1* kinase domain hotspot missense mutations (p.N546 or p.K656) in combination with *PIK3CA* or *PIK3R1* mutation. These tumors are characterized by a glial component typically with oligodendroglial morphology together with an admixed neurocytic component consisting of neurocytic rosettes and/or perivascular neuropil, frequently in a mucin-rich stroma

The 10 patients with *FGFR1*-altered LGNET with DNA methylation profiles aligning to RGNT ranged from 11 to 46 years of age at time of initial diagnosis (median 21.5 years) and included 4 males and 6 females. Eight tumors were located in the 4th ventricle or cerebellum, and two tumors were located in the third ventricle. Seven tumors were histologically consistent with rosette-forming glioneuronal tumor, whereas three tumors showed histologic features of a low-grade oligodendroglial tumor NOS (not otherwise specified) without well-defined neurocytic rosettes on either H&E or synaptophysin staining (representative histology shown in Fig. 3).

All ten tumors harbored a hotspot missense mutation within exons encoding the tyrosine kinase domain of *FGFR1*, resulting in either an asparagine to lysine substitution at codon 546 (p.N546K, n = 7) or a lysine to glutamic acid substitution at codon 656 (p.K656E, n = 3). Two of these tumors harbored an additional missense mutation in *FGFR1* (p.K523T and p.D652G), both of which were present in cis (on the same allele) as the respective hotspot mutation. Four of the tumors (RGNT #2, #6, #7, and #9) harbored copy-neutral loss of heterozygosity of chromosome 8p containing the *FGFR1* locus, resulting in two copies of the mutant *FGFR1* allele being present. Additionally, one other tumor (RGNT #5) harbored trisomy of chromosome 8 containing the *FGFR1* locus, resulting in two copies of the mutant *FGFR1* allele being present.

In addition to *FGFR1* mutation, nine of the ten tumors additionally harbored mutually exclusive mutations in either *PIK3CA* or *PIK3R1*, with seven containing activating mutations in the *PIK3CA* catalytic subunit and two containing inactivating small in-frame deletions in the *PIK3R1* negative regulatory subunit. Accounting for the impact of trisomy 8 or loss of heterozygosity involving chromosome 8p, the relative mutant allele frequencies of *FGFR1* versus *PIK3CA* or *PIK3R1* were approximately equivalent in 7 of the 9 tumors, indicating that these two events were both likely to have occurred early during tumor evolution and were present clonally throughout all tumor cells. However, in two tumors (RGNT #4 and #7), the *FGFR1* mutant allele frequency was appreciably higher than the *PIK3CA*/*PIK3R1* mutant allele frequency (beyond that explained by the loss of heterozygosity of chromosome 8p alone in RGNT #7), indicating that the *FGFR1* mutation arose before the *PIK3CA* or *PIK3R1* mutation during the clonal evolution of these two tumors. Of the seven tumors with dual *FGFR1* + *PIK3CA* mutations, four contained accompanying *NF1* mutations (one frameshift, two missense, and one small in-frame deletion). Of the two tumors with dual *FGFR1 *+ *PIK3R1* mutations, one contained an accompanying *PTPN11* mutation (p.A72T), which is a known mutational hotspot in myeloid neoplasms thus providing support for pathogenicity [Catalog of Somatic Mutations in Cancer database v91 release]. Only one of the 10 epigenetically confirmed RGNT harbored *FGFR1* mutation without an accompanying *PIK3CA* or *PIK3R1* mutation or any other accompanying likely pathogenic alterations.

### Pilocytic astrocytoma

**Fig. 4:**
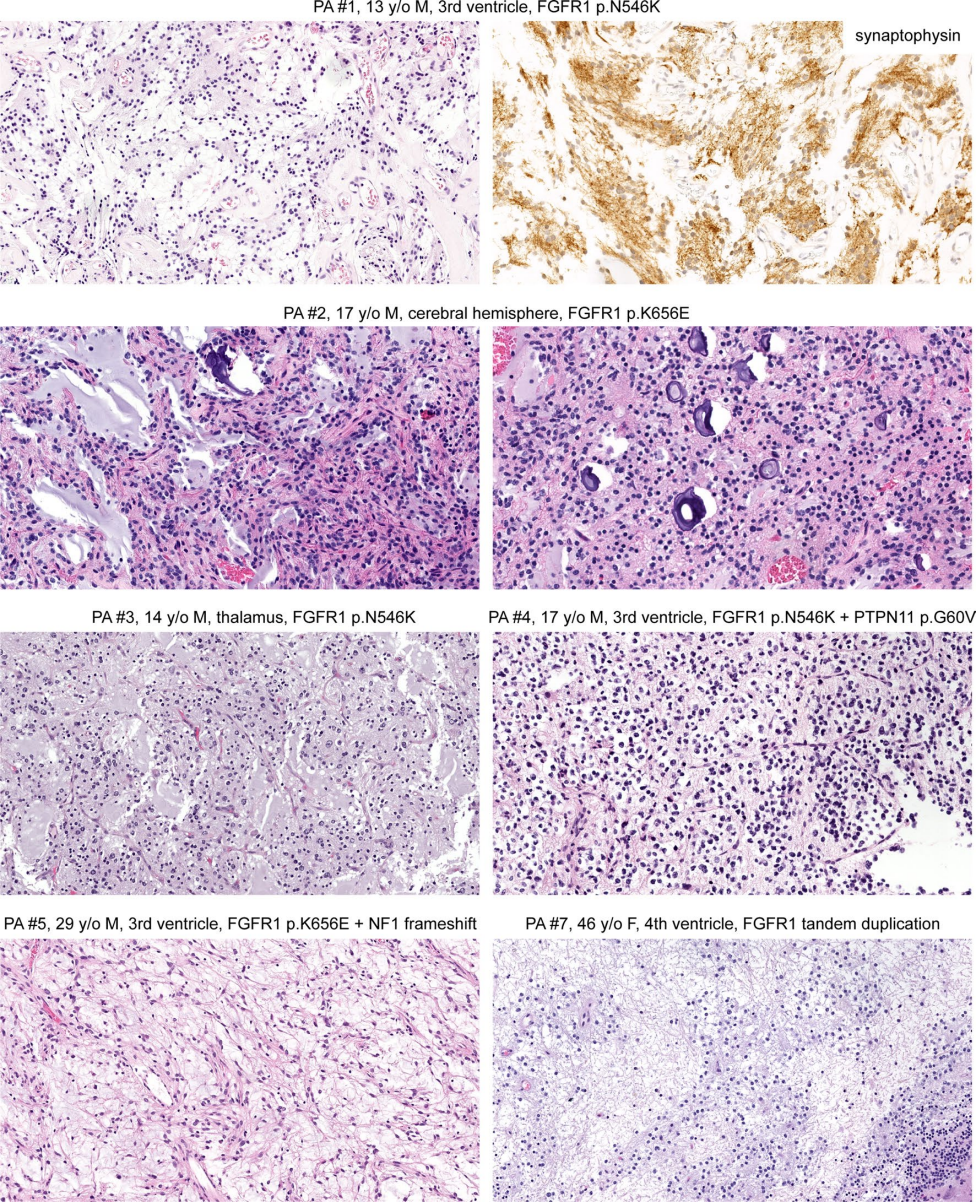
Histologic spectrum of epigenetically defined pilocytic astrocytomas harboring *FGFR1* alterations. These tumors are characterized by a glial component with predominantly oligodendroglial morphology often in a mucin-rich stroma with variable presence of microcalcifications. Occasional tumors demonstrate neurocytic differentiation and neurocytic rosettes resembling RGNT (case PA #1). In contrast to pilocytic astrocytomas with *BRAF* mutation or fusion that typically have piloid morphology, frequent Rosenthal fibers, and biphasic pattern of alternating loose and compact growth, these pilocytic astrocytomas with *FGFR1* alterations typically have an oligodendroglial morphology in a prominent myxoid stroma without Rosenthal fibers

The eight patients with *FGFR1*-altered LGNET with DNA methylation profiles aligning to pilocytic astrocytoma ranged from 10 to 72 years of age at time of initial diagnosis (median 17 years) and included 6 males and 2 females. Three tumors were located in the third ventricle, one in the fourth ventricle/cerebellum, one in the cerebral hemispheres, one in the lateral ventricle, one in the thalamus, and one in the spinal cord. The histologic spectrum of these eight tumors was variable, and included four cases composed of bipolar glioma cells with piloid processes resembling conventional pilocytic astrocytoma, albeit lacking appreciable Rosenthal fibers (representative histology shown in Fig. 4). The other four tumors included PA #1 that contained numerous neurocytic rosettes resembling rosette-forming glioneuronal tumor, and PA #3 that displayed variable areas of both piloid and oligodendroglial morphology with a single focus containing well-formed neurocytic rosettes. The remaining two tumors (PA #4 and PA #7) were composed of a low-grade proliferation of glial cells with oligodendroglial morphology in a prominent myxoid stroma but without well-defined patterned nodules, floating neurons, neurocytic rosettes, piloid process, Rosenthal fibers, or other specific histologic findings.

Three of the eight tumors harbored tandem duplication of the 3′ exons encoding the tyrosine kinase domain of *FGFR1*, and the remaining five tumors harbored hotspot missense mutations within the tyrosine kinase domain of *FGFR1* (p.N546K, n = 3; p.K656E, n = 2). Two of these five tumors harbored an additional missense mutation in *FGFR1* (p.R675G and p.V561M), both of which were too distant to phase from their respective hotspot mutations. Among the five tumors with *FGFR1* kinase domain hotspot missense mutations, one harbored an accompanying *NF1* frameshift mutation and one harbored an accompanying *PTPN11* hotspot missense mutation (p.G60V), which is a known pathogenic variant. None of the three tumors with *FGFR1* kinase domain tandem duplication were identified to have any additional likely pathogenic alterations, including the *PIK3CA*, *PIK3R1*, *NF1*, and *PTPN11* genes.

### Dysembryoplastic neuroepithelial tumor

**Fig. 5:**
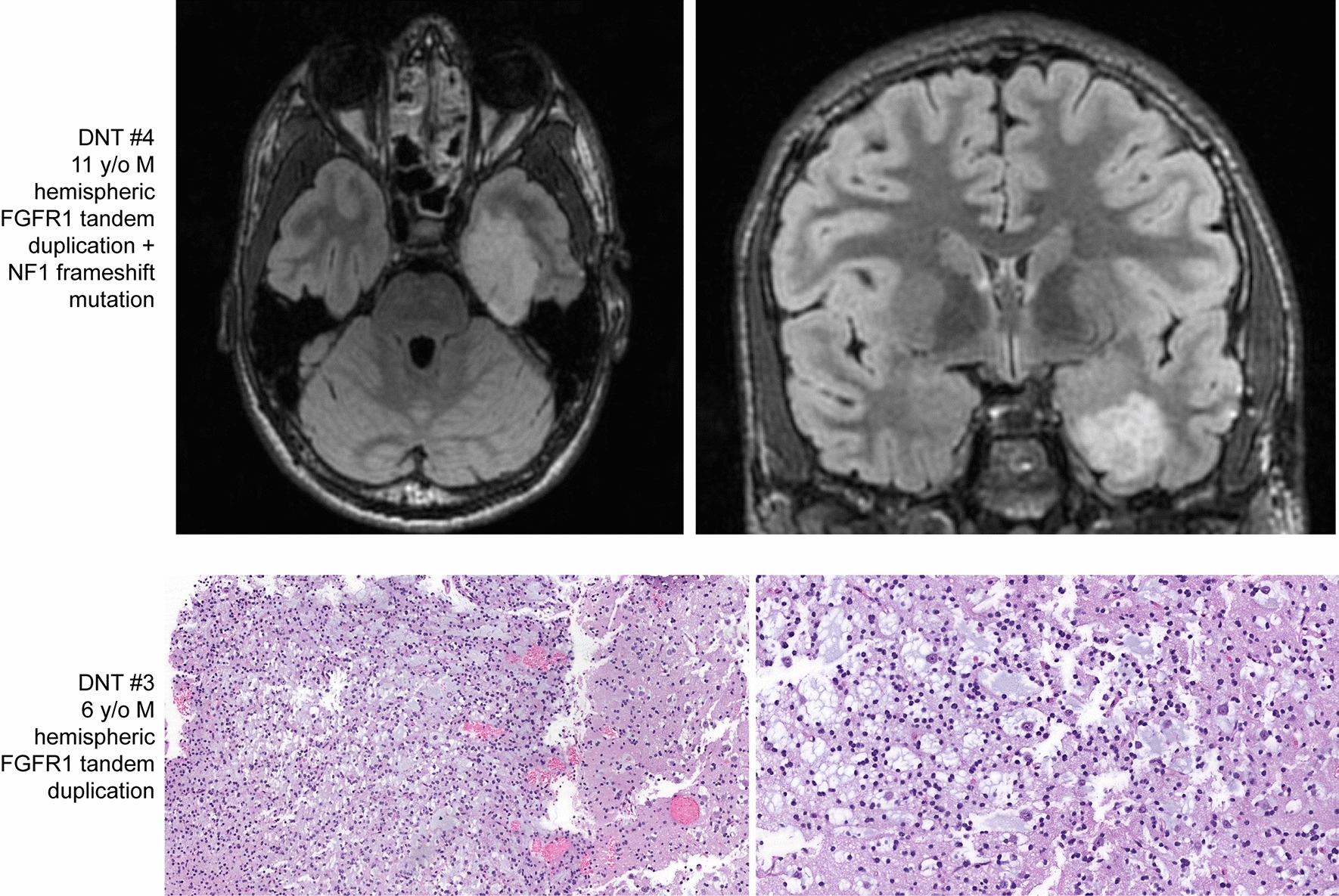
Imaging and histologic features of epigenetically defined dysembryoplastic neuroepithelial tumors (DNT) harboring *FGFR1* alteration. These tumors demonstrate classic histology of DNT including mucin-rich patterned nodules, oligodendrocyte-like glial component, and floating neurons within the mucinous stroma

The five patients with *FGFR1*-altered LGNET with DNA methylation profiles aligning to DNT ranged from 6 to 46 years of age at time of initial diagnosis (median 22 years) and included 3 males and 2 females. Four tumors were located in the cerebral hemispheres, and one was centered in the lateral ventricle. One tumor demonstrated histologic features that were prototypical for DNT, with mucin-rich patterned nodules composed of oligodendroglial tumor cells with admixed floating neurons (representative histology shown in Fig. 5). The other four tumors showed histologic features of a low-grade oligodendroglial tumor NOS with prominent myxoid stroma but without well-defined patterned nodules or floating neurons.

Three of the five tumors harbored tandem duplication of the 3′ exons encoding the tyrosine kinase domain of *FGFR1*, and the remaining two tumors harbored hotspot missense mutations within the tyrosine kinase domain of *FGFR1*. Interestingly, DNT #1 harbored two hotspot mutations in *FGFR1* (both p.N546S and p.K656E, present at 33% and 38% allele frequencies, respectively) but which were too distant to phase. Notably, almost all mutations at codon 546 in the *FGFR1* gene in glial and glioneuronal tumors have been either p.N546K or p.N546D, and the p.N546S substitution in this DNT is rare but also likely to be activating. DNT #2 harbored the p.N546K hotspot mutation in *FGFR1* and also had an accompanying *PTPN11* hotspot missense mutation (p.G503V), which is a known pathogenic variant. Two of the three tumors with *FGFR1* kinase domain tandem duplications harbored accompanying *NF1* mutations (one frameshift and one missense).

### Extraventricular neurocytoma

**Fig. 6:**
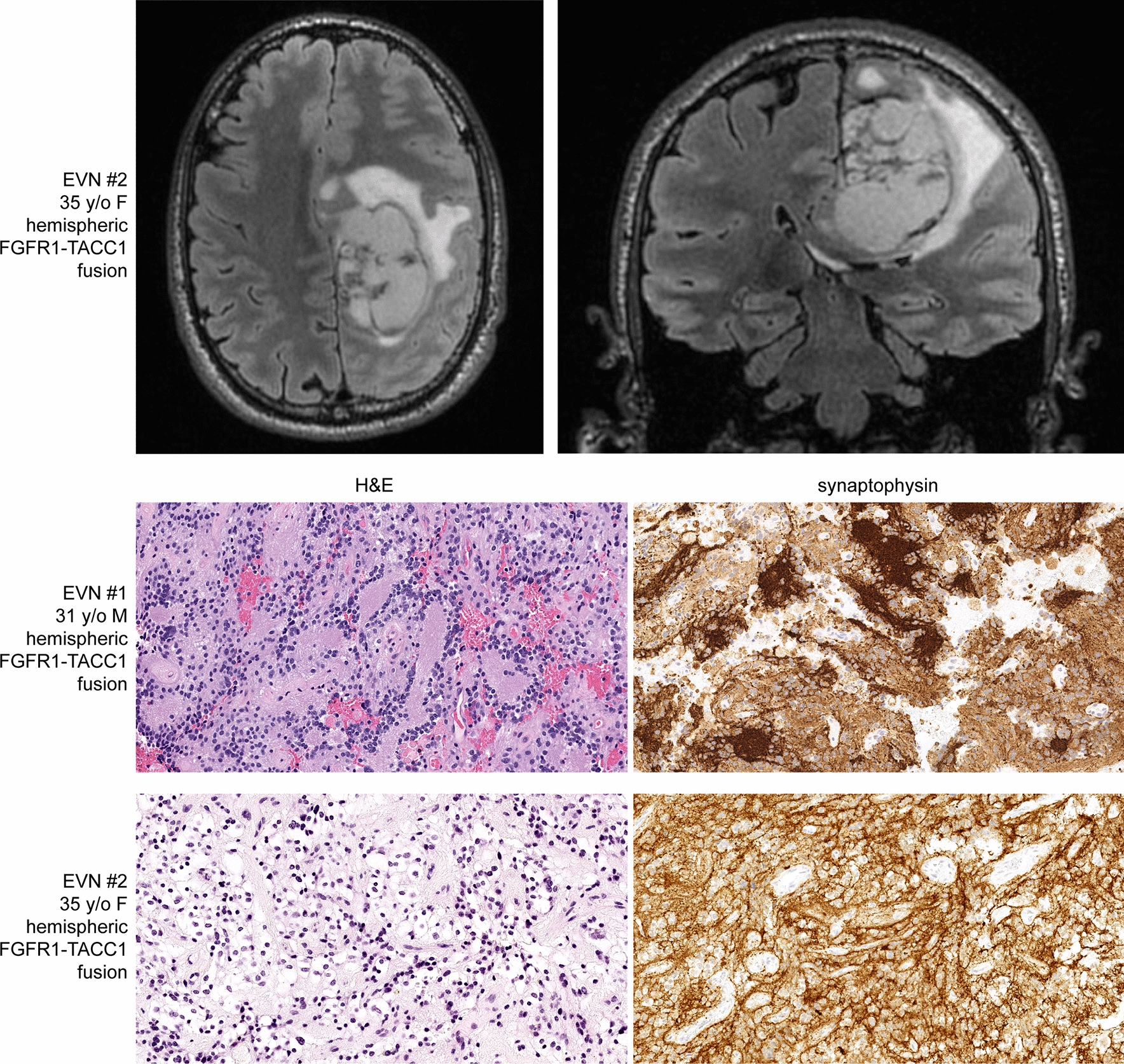
Imaging features and histologic spectrum of epigenetically defined extraventricular neurocytomas harboring *FGFR1*-*TACC1* fusion. These tumors are characterized by a low-grade proliferation of neurocytic cells forming rosettes around central neuropil cores with diffuse strong synaptophysin positivity of the tumor cells and neuropil stroma

The two patients with *FGFR1*-altered LGNET with DNA methylation profiles aligning to EVN were both males and had ages of 31 and 35 years at time of diagnosis. Both tumors were located in the cerebral hemispheres and not connected with the ventricular system. Both tumors were histologically composed of neurocytic cells forming numerous neurocytic rosettes without an admixed glial component and had diffuse strong synaptophysin expression (representative histology shown in Fig. 6). Both tumors harbored an in-frame *FGFR1*-*TACC1* gene fusion, consisting of exons 1-18 (EVN #1) or exons 1-17 (EVN #2) of *FGFR1* fused to exons 7-13 of *TACC1*. Besides the *FGFR1*-*TACC1* fusion, no other additional likely pathogenic alterations were identified, including the *PIK3CA*, *PIK3R1*, *NF1*, and *PTPN11* genes.

### Unclassifiable low-grade neuroepithelial tumors

Five patients with *FGFR1*-altered LGNET had DNA methylation profiles that did not align with any reference methylation classes by either UMAP clustering analysis or random forest classification using the DKFZ MolecularNeuropathology.org online classifier tool (version 11b4). Notably, the DNA methylation profiles of these five tumors did not cluster together, suggesting that they do not represent a single new tumor type. Rather, the epigenetic signature of uLGNET #1 was distant from all reference CNS tumor entities included in the UMAP clustering analysis except for one divergent tumor assigned to the PA, PF reference cohort (GEO sample ID: GSM2403459). The epigenetic signature of uLGNET #2–5 was closest to DNT and PA, ST reference methylation classes, the significance of which remains unclear in terms of reliable diagnostic classification.

**Fig. 7:**
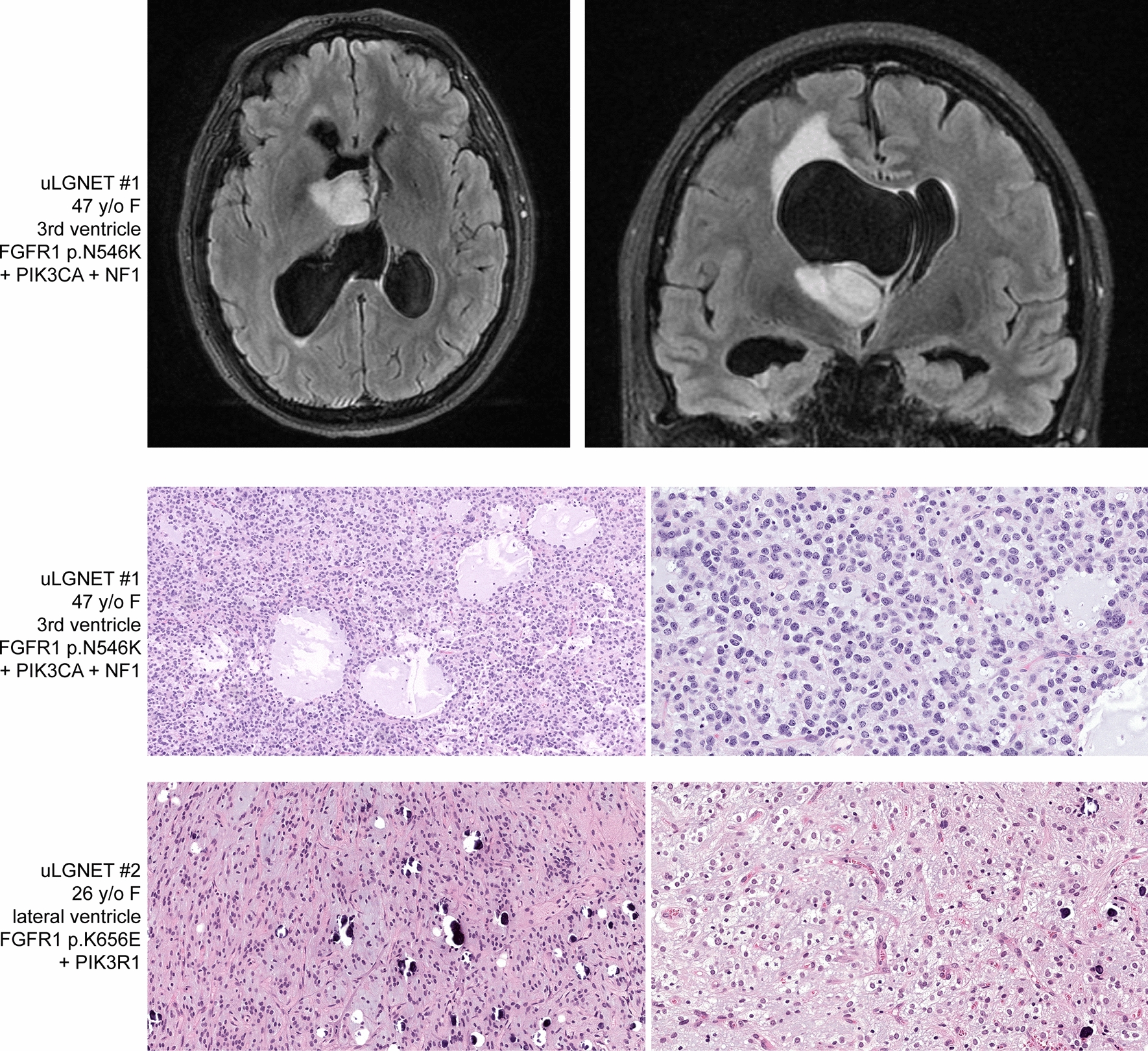
Imaging features and histologic spectrum of unclassifiable LGNET harboring *FGFR1* alterations. These tumors are unlikely to represent a unified group, and may likely represent either novel rare glioma subtypes or tumors that did not reliably cluster by methylation analysis due to low tumor content or other technical issues. Patient uLGNET #1 is a 47 year old woman with a heterogeneously enhancing, T2 hyperintense mass centered in the third ventricle. Histology demonstrated a low-grade glial neoplasm with solid growth pattern, mucin-rich stroma with frequent microcysts, and rare mitotic figures. Patient uLGNET #2 is a 26 year old female with a heterogeneously enhancing mass centered in the posterior horn of the lateral ventricle that was histologically composed of a low-grade oligodendroglial neoplasm with frequent microcalcifications and mucin-rich stroma, but lacked any well-defined neurocytic rosettes

The five patients with *FGFR1*-altered LGNET with unclassifiable DNA methylation profiles ranged from 8 to 47 years of age at time of initial diagnosis (median 26 years) and included 1 male and 4 females. Two tumors were located in the lateral ventricles, two in the third ventricle, and one in the cerebral hemispheres. These five unclassifiable tumors were composed of a low-grade proliferation of glial cells with oligodendroglial morphology in a prominent myxoid stroma but without well-defined patterned nodules, floating neurons, neurocytic rosettes, piloid process, Rosenthal fibers, or other specific histologic findings (representative histology shown in Fig. 7).

One unclassifiable LGNET harbored *FGFR1* kinase domain tandem duplication as the solitary pathogenic alteration identified, whereas the other four all harbored hotspot missense mutations within the tyrosine kinase domain of *FGFR1* (p.N546K, n = 1; p.K656E, n = 3). The one unclassifiable LGNET with *FGFR1* p.N546K mutation harbored additional *PIK3CA* p.G118D missense mutation and an intragenic deletion involving exon 35 of the *NF1* gene predicted to disrupt gene function. One of the three unclassifiable LGNET with *FGFR1* p.K656E mutation harbored an accompanying *PIK3R1* p.K567E missense mutation, which is a specific variant that has been recurrently found in gliomas and other tumor types [Catalog of Somatic Mutations in Cancer database v91 release]. The three unclassifiable LGNET with *FGFR1* p.K656E each harbored additional missense mutations in *FGFR1* (p.D652G, n = 2; p.I544V, n = 1). These additional *FGFR1* missense mutations were present in cis when phasing could be evaluated (two of the three tumors).

## Discussion

**Fig. 8:**
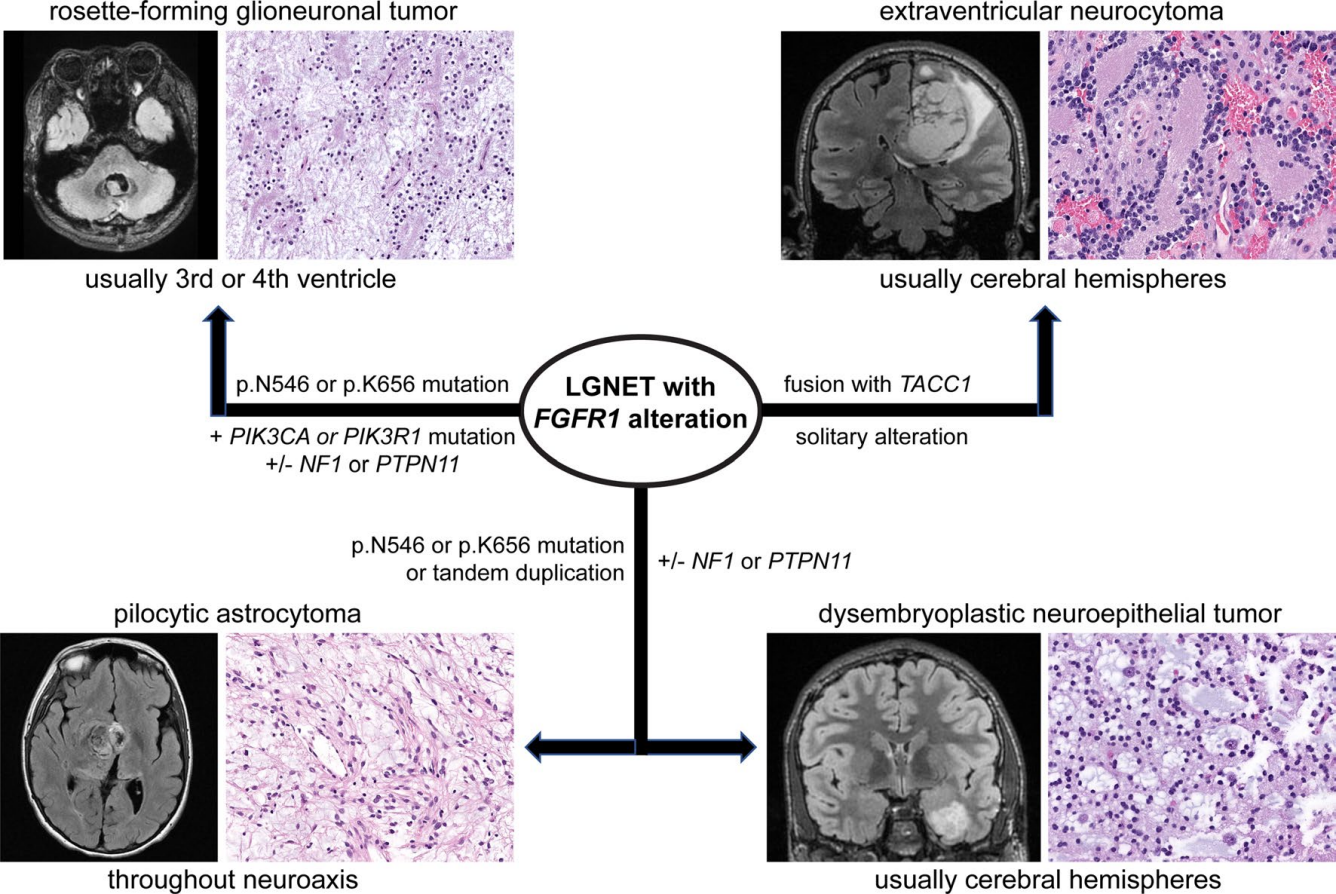
Low-grade neuroepithelial tumors with *FGFR1* alterations are best stratified based on a combination of tumor location, histologic features, specific *FGFR1* alteration, accompanying genetic alterations, and epigenetic signature

Through combining histologic, genomic, and epigenetic profiling on a series of 30 LGNET with *FGFR1* alterations, we have identified that some tumor entities (RGNT and EVN) have a distinct pattern of genetic alterations, while other tumor entities (pilocytic astrocytoma and DNT) have overlapping/indistinct patterns precluding accurate classification based solely on genetic aberrations (Fig. 8). Epigenetically confirmed RGNT harbor either p.N546 or p.K656 hotspot missense mutations in the kinase domain of *FGFR1*, and do not have *FGFR1* kinase domain tandem duplication or gene fusions [36]. Additionally, the vast majority of RGNT have accompanying, mutually exclusive mutations in either *PIK3CA* or *PIK3R1*, predicted to cause activation of the PI3-kinase-Akt-mTOR signaling pathway, along with additional *NF1* or *PTPN11* mutations in a subset [11, 36]. While pilocytic astrocytoma and DNT can also harbor identical hotspot missense mutations in *FGFR1*, the combination of co-occurring *FGFR1* p.N546 or p.K656 mutation together with either *PIK3CA* or *PIK3R1* mutation in LGNET appears to be specific for RGNT and was not found in any epigenetically confirmed cases of pilocytic astrocytoma, DNT, and EVN in this cohort or the published literature to date [38, 40]. Moreover, our study confirms that epigenetically confirmed EVN is characterized by frequent *FGFR1*-*TACC1* gene fusion [38], which is rare or absent in other LGNET subtypes including RGNT, DNT, pilocytic astrocytoma, and ganglioglioma both in this cohort and previous studies [29, 31, 33, 34, 36, 44].

DNA methylation profiling is now an important ancillary diagnostic tool to refine classification of human neoplasia. In terms of low-grade glial and glioneuronal tumors, prior studies have demonstrated that RGNT, EVN, and DNT each group into a single distinct methylation cluster, whereas pilocytic astrocytomas segregate into three distinct methylation clusters (which have been designated supratentorial, midline, and posterior fossa) [7, 21]. In agreement with *FGFR1* alterations being promiscuously found amongst a wide spectrum of different CNS tumor types, *FGFR1*-altered LGNET do not form a distinct methylation cluster. Rather, the majority of *FGFR1*-altered LGNET are epigenetically dispersed amongst the RGNT, EVN, DNT, and three different pilocytic astrocytoma methylation clusters. While the RGNT, EVN, and DNT methylation clusters are composed of tumors with nearly universal *FGFR1* alterations, the three pilocytic astrocytoma methylation clusters are composed of a mixture of tumors with alterations in various genes causing activation of the MAP kinase signaling pathway (*e.g. BRAF*, *FGFR1*, *NF1*, *RAF1*, *KRAS*) [7, 33, 36, 38]. Notably, the three distinct methylation clusters of pilocytic astrocytoma segregate primarily based on tumor location (supratentorial, midline, and posterior fossa) and not by underlying genetic alteration. Accordingly, we document examples of pilocytic astrocytomas with *FGFR1* alterations located throughout the neuroaxis and belonging to each of the three different methylation classes.

Here we demonstrate that the majority of LGNET with *FGFR1* alterations, both pediatric and adult, epigenetically cluster with the previously defined reference DNA methylation classes. However, a subset of cases (5/30, 17%) did not closely cluster with any reference methylation classes. The potential explanations for this are multiple. First is that the relative tumor content of these samples is low, which was the case for two of the five tumors (uLGNET #3 and #4) based on the low *FGFR1* mutant allele frequencies of 10% and 6%, respectively. The remaining three tumors had high tumor content based on *FGFR1* mutant allele frequencies of > 25%, indicating that the reason for failure to closely cluster was due to other causes. Two other possibilities are: (1) representing novel tumor entities not represented in the reference cohort used for clustering analysis, and (2) representing defined tumor entities but with biologic variation in epigenetic signature beyond the group of tumors composing the specific reference methylation class. Notably, two of the unclassifiable LGNET with high tumor content (uLGNET #1 and #2) had genetic signatures of RGNT with *FGFR1* kinase domain hotspot missense mutation in combination with *PIK3CA* or *PIK3R1* mutation. Neither of these tumors were located in the stereotypic location for RGNT in the fourth ventricle, with one tumor located in the anterior third ventricle and the other located in the posterior horn of the left lateral ventricle, and neither displayed well-formed neurocytic rosettes on histology. Whether these two tumors represent true RGNT and have epigenetic signatures as part of a yet-to-be defined methylation class of RGNT outside of the stereotypic location in the fourth ventricle is a distinct possibility (similar to how pilocytic astrocytoma has three distinct methylation classes based on tumor location).

It is now well appreciated that classification of low-grade glial and glioneuronal tumors based on histologic features alone can be challenging [40]. While some CNS neoplasms demonstrate classic histologic features of a specific tumor entity and do not necessarily require ancillary molecular testing for diagnostic confirmation, a significant subset of low-grade glial and glioneuronal tumors demonstrate ambiguous or overlapping morphologic features precluding reliable diagnosis based on histologic features alone necessitating ancillary molecular testing. However, the most efficient and accurate method for CNS tumor classification remains uncertain, be it combining histology with targeted next-generation sequencing versus DNA methylation profiling or other methodologies. Notably, this cohort of *FGFR1*-altered LGNET included many tumors that were difficult to accurately classify based on histologic features alone. As has been previously reported for *FGFR1*-altereted LGNET, the vast majority of the tumors in this series demonstrated an oligodendroglial morphology of the glial component and a prominent myxoid stroma [31, 37]. This included the majority of the pilocytic astrocytomas, which often lacked the prototypical Rosenthal fibers, piloid processes, and biphasic pattern with alternating loose and compact growth that is characteristic of pilocytic astrocytomas with *KIAA1549*-*BRAF* fusion.

Remarkably, three of the tumors in this cohort with intraventricular location that had both genetic and epigenetic profiles aligning with RGNT did not have well-defined neurocytic rosettes on either H&E or synaptophysin staining and were best characterized as low-grade oligodendroglial neoplasm NOS based on histologic features. In such cases, ancillary genomic evaluation or DNA methylation profiling can be informative with definitive tumor classification. Also noteworthy is two tumors (PA #1 and PA #3) that had histologic features of RGNT with well-defined neurocytic rosettes containing synaptophysin-positive neuropil cores either diffusely (PA#1) or focally (PA #3). Both of these tumors lacked the accompanying *PIK3CA* or *PIK3R1* mutation that is characteristic of RGNT, and both had epigenetic profiles aligning with pilocytic astrocytoma rather than RGNT. The true nature and best classification for such tumors with RGNT-like histology but molecular features aligning with pilocytic astrocytoma remains uncertain.

Together with the prior study by Sievers et al. [36], our findings further confirm that RGNT is a unique glioneuronal tumor type with both a distinct epigenetic signature and distinct combination of *FGFR1* hotspot missense mutation together with mutually exclusive mutation of either *PIK3CA* or *PIK3R1*, the latter of which is a novel finding of this study. Notably, a subset of RGNT (4/10, 40%) have copy-neutral loss of heterozygosity involving chromosome 8p that eliminates the wildtype allele and results in two copies of the mutant *FGFR1* allele in tumor cells. This is a unique finding for *FGFR1* and RGNT, as there is not typically selection pressure for loss of the remaining wildtype allele for other mutated oncogenes in human cancers of any other type (*e.g. KRAS*, *BRAF*, *EGFR*) [43]. Comparing the relative *FGFR1* and *PIK3CA*/*PIK3R1* mutant allele frequencies in RGNT when accounting for the impact caused by trisomy 8 or loss of heterozygosity involving chromosome 8p, we find that the *FGFR1* mutation is uniformly clonal and present in all tumor cells as an early or initiating event, whereas the *PIK3CA* or *PIK3R1* mutations either arise at a similar timepoint or occasionally as a later event during tumor evolution after *FGFR1* mutation.

Interestingly, the majority of RGNT have at least two, and often three, different pathogenic mutations involving *FGFR1*, either *PIK3CA* or *PIK3R1*, and often also *NF1* or *PTPN11*. As such, genetic activation of both the Ras-Raf-MEK-ERK and PI3-kinase-Akt-mTOR signaling pathways appears to be fundamental to the pathogenesis of RGNT. This underlying pathogenesis of RGNT is different than other low-grade glioneuronal tumors such as ganglioglioma and pilocytic astrocytoma that typically involve a solitary pathogenic alteration (*e.g. BRAF* mutation or fusion) causing activation of the MAP kinase signaling in isolation [18, 29, 34, 44]. Why RGNT are low-grade neoplasms with mostly indolent behavior despite the selection for and acquisition of multiple pathogenic mutations involving two mitogenic signaling pathways remains unknown, but may involve having intact cell cycle regulators such as *CDKN2A* and absence of telomere maintenance mechanism (e.g. *TERT* promoter mutation or *ATRX* inactivation) that protect against uncontrolled proliferation and malignant transformation.

In summary, we show that *FGFR1* alterations occur in a wide spectrum of known tumor entities with overlapping histologic features. Integrating the pattern of genetic alterations and/or epigenetic signature for such low-grade glial and glioneuronal tumors can assist with accurate diagnostic classification and prognostication for affected patients.

## Supplementary information

**Additional file 1: Tables S1–S6.**

## Data Availability

Scanned image files of H&E stained sections and select synaptophysin stained sections from 26 of the tumors in this cohort are available for downloading and viewing at the following link: https://figshare.com/projects/Low-grade_neuroepithelial_tumors_with_FGFR1_alterations/81740. DNA methylation array data files from this study are available from the Gene Expression Omnibus (GEO) repository under accession number GSE152653 (https://www.ncbi.nlm.nih.gov/geo/). Mutation, structural variant, and copy number data are available in the electronic supplementary material. Raw sequencing data files are available from the authors upon request.
